# Liver steatosis and fibrosis in acromegaly patients with active and controlled disease: a scoping review

**DOI:** 10.1007/s40618-026-02940-1

**Published:** 2026-06-04

**Authors:** Karol P. Sagan, Zuzanna Lipiec-Rzepecka, Martyna Patalong-Wójcik, Michał P. Wasilewicz, Elżbieta Andrysiak-Mamos, Anhelli Syrenicz

**Affiliations:** 1 Department of Endocrinology, Metabolic and Internal Diseases, University Clinical Hospital no. 1, Pomeranian Medical University, Szczecin, Poland; 2 Department of Hepatology and Liver Transplantology, University Clinical Hospital no. 1, Pomeranian Medical University, Szczecin, Poland

**Keywords:** Acromegaly, Liver steatosis, Liver fibrosis, Intrahepatic lipid content, Hepatic steatosis

## Abstract

**Purpose:**

Excess growth hormone, as encountered in acromegaly, promotes insulin resistance and enhances adipose tissue lipolysis. This study aimed to summarize the currently available data on the prevalence of hepatic steatosis and liver fibrosis in patients with acromegaly. The secondary objective was to map the studies on the influence of treatment modalities on hepatic outcomes in this population.

**Methods:**

This scoping review was conducted following the PRISMA-ScR guidelines. We performed a systematic literature search across two electronic databases: PubMed and Web of Science from their inception to the 4th of April 2026. In addition, articles were identified through manual searches using Google Scholar and citation tracking. Peer-reviewed original journal articles evaluating hepatic steatosis, fibrosis, and intracellular hepatic lipid (IHL) content in patients with acromegaly were included. Studies that did not utilize radiological methods and case reports were excluded. Results were synthesized narratively and summarized in tables.

**Results:**

Twenty-one original studies were included. High heterogeneity was observed among the studies, including study models, imaging methods, patient populations, and outcomes. The data presented in this literature review reveal a pattern of lower IHL content in active acromegaly patients but a higher likelihood of liver fibrosis compared with controls. Control of acromegaly may be associated with a rise in IHL content.

**Conclusions:**

IHL content and hepatic fibrosis in patients with acromegaly may be influenced by disease control status and treatment modalities. Further high-quality studies are required to assess the risk of liver steatosis and fibrosis in this patient population.

## Introduction

Acromegaly is a rare endocrinological disorder caused by excess growth hormone (GH) and insulin growth factor-I (IGF-I). It leads to metabolic, cardiovascular, musculoskeletal, and neoplastic complications [1]. Depending on the study population the prevalence of acromegaly varies between 2.8 and 13.7 cases per 100,000 individuals [2].

The hormonal profile of active acromegaly (AA) induces peripheral insulin resistance and promotes lipolysis of subcutaneous and visceral adipose tissue, thus leading to increased levels of circulating free fatty acids. These mechanisms promote ectopic lipid accumulation, defined as the deposition of excess triglycerides in non-adipose tissues that physiologically contain minimal fat [3]. A recent systematic review demonstrated increased lipid accumulation in the skeletal muscle of acromegaly patients [4].

Hepatic steatosis is defined as accumulation of lipids in at least 5% of liver cells [5]. It develops when the hepatic supply exceeds its capacity to process lipids, either due to increased dietary intake or enhanced lipolysis of adipose tissue and visceral fat. This process is amplified by insulin resistance in adipocytes [6]. Under physiological conditions, hepatic lipogenesis is counterbalanced by the export of triglycerides through the secretion of very low density lipoprotein (VLDL) from the liver into the circulation. Progression from steatosis to fibrosis in some individuals has been explained by the multiple-parallel hit hypothesis [7].

Steatotic liver disease (SLD) is a term that encompasses a broad spectrum of clinical entities characterized by hepatic steatosis. It represents a growing public health care problem, with a global prevalence estimated at approximately 38% [8]. In the group of overweight and obese patients, this percentage is even higher, estimated at 70% and 75%, respectively [9]. SLD is associated with an increased risk of hepatic and extrahepatic cancer, as well as cardiovascular and all-cause mortality [10]. However, it remains largely underdiagnosed [11]. In June 2023, an expert panel approved a new classification and terminology, separating subcategories of SLD based on dominant risk factors [10]. The current terms metabolic dysfunction-associated steatotic liver disease (MASLD) and metabolic dysfunction-associated steatohepatitis (MASH) correspond to non-alcoholic fatty liver disease (NAFLD) and non-alcoholic steatohepatitis (NASH), respectively [12].

The existing literature on liver steatosis and fibrosis in acromegaly is heterogeneous and inconclusive and the relationship between acromegaly and SLD has not yet been fully elucidated. Current evidence indicates that the traditional risk factors for hepatic steatosis may be of limited relevance in the context of acromegaly.

In this study we aimed to summarize the available data in acromegaly regarding:

1) the prevalence of hepatic steatosis and intracellular hepatic lipid (IHL) content,

2) the prevalence of liver fibrosis,

3) the association between liver steatosis, IHL content and liver function,

4) the influence of treatment modalities on IHL content, liver steatosis and function.

According to our knowledge no other scoping review on this topic has been published. We adopted the nomenclature of SLD originally used in the cited studies.

## Methods

### Search strategy and protocol

This scoping review was conducted following the PRISMA-ScR guidelines [13] and has not been registered.

A comprehensive search was performed across two electronic databases: PubMed and Web of Science from their inception to the 4th of April 2026 utilizing the following term: acromegaly AND (“NAFLD” OR “MAFLD” OR “MASLD” OR “fatty liver” OR “steat* liver” OR “fibro* liver” OR “intrahepatic lipid”). In addition, articles were identified through manual searches using Google Scholar and citation tracking. No language restrictions were imposed. Peer-reviewed original journal articles evaluating hepatic steatosis, fibrosis, and IHL content in patients with acromegaly were included. Studies that did not utilize radiological methods and case reports were excluded. Duplicates were manually removed. Two authors independently screened title and abstracts, followed by a full-text review of potentially relevant articles. Reasons for exclusion were documented, and discrepancies between authors were resolved by consensus. Two reviewers developed a table, which was updated and modified during the literature analysis process. Data on study model, patient body mass index (BMI), prevalence of diabetes, liver examination technique, types of medical interventions, follow-up duration, and clinical outcomes were manually extracted and summarized in tables independently by two authors. We resolved disagreements on data extraction by consensus during discussion which involved a third independent author. The included studies were grouped into five categories: prevalence of liver steatosis, IHL content, prevalence of hepatic fibrosis, association of liver steatosis and liver function, influence of treatment modalities on liver steatosis and function.

## Results

**Fig. 1:**
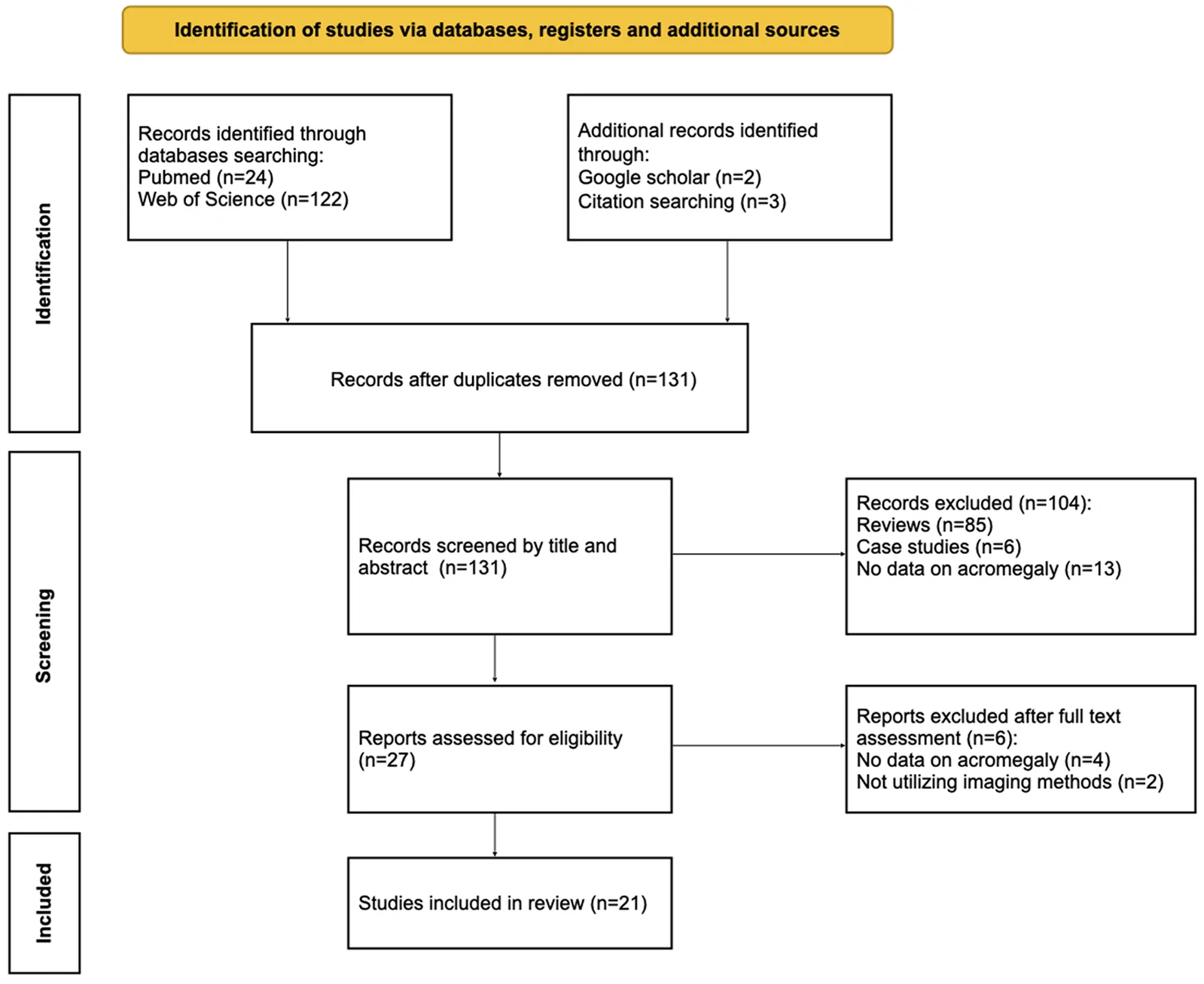
Flow diagram for the screening review process

A literature search of PubMed and Web of Science databases resulted in one hundred twenty-six citations. Additionally, five articles were identified as eligible based on manual citation tracking and Google Scholar search. Ultimately, twenty-one studies were included in the analysis [14–34]. Figure 1 shows a flow diagram of the identification, screening, and inclusion processes.

### Types of studies identified

Of the twenty-one studies included, eleven had a cross-sectional design, six had a combined cross-sectional and prospective longitudinal design, three were longitudinal prospective study and one was a retrospective case-control study. Ultrasonography (USG) was utilized to assess liver steatosis or fibrosis in nine studies [14–22], while twelve studies employed magnetic resonance imaging (MRI) to evaluate intracellular hepatic lipid (IHL) content or presence of steatotic liver disease (SLD) [23–34].

### Patients stratification and control populations

The criteria used to define controlled diseases were inconsistent among the identified manuscripts. Some authors defined patients as hormonally controlled only by insulin growth factor-I (IGF-I) levels below the upper limit of normal adjusted for age [14, 23–26] or ≤ 1,2 the upper limit of normal adjusted for age [27]. Some authors required adjusted IGF-I levels and growth hormone (GH) suppression in the oral glucose tolerance test (OGTT) [22, 28], or a random GH concentration below 0.5 µg/L [29] or below 1 µg/L [15, 16]. One study defined biochemical control as GH suppression below 1 ng/mL during the OGTT without requirements regarding IGF-I levels [30]. Some authors did not clearly define the criteria based on which patients were classified as having controlled acromegaly (CA) [17, 31, 32].

The exclusion criteria differed among the studies. Four of the twenty-one studies excluded acromegaly patients with diabetes [16, 26, 30, 31], one excluded individuals taking insulin [23], one excluded patients with uncontrolled diabetes and those receiving insulin therapy [18]. One study excluded patients taking medication known to impact hepatic steatosis, e.g. glucagon like peptide-1 receptor agonist and pioglitazone [22]. Seven studies excluded patients with a history of alcohol abuse [14, 15, 18–20, 22, 23]. Most studies excluded patients previously diagnosed with liver disease. Fourteen studies included a control group [15, 17, 18, 20–24, 27, 28, 30–33]. The control groups were selected among “healthy” and “non-acromegalic” individuals matched for several criteria such as age, sex, BMI. In one study two control groups were included: one consisted of metabolic-, age- and sex-matched individuals, the second included metabolically healthy sex-matched individuals [22].

### Prevalence of liver steatosis in patients with acromegaly and intergroup comparisons

The prevalence of liver steatosis in patients with acromegaly was assessed using imaging techniques in ten studies [14–23], most of which had a cross-sectional design. USG was the most commonly used method, used in nine studies [14–22]. Additionally, in four of these nine studies quantitative USG techniques were applied: controlled attenuation parameter (CAP) [14, 20, 22] and tissue attenuation imaging (TAI) [18]. Only one study utilized MRI proton density fat fraction (MRI-PDFF) [23] to assess SLD.

In summary, the prevalence of SLD ranged from 0% to 71% and from 30% to 92% in patients with active acromegaly (AA) and CA, respectively. For comparison, in the control groups, the prevalence of SLD ranged from 18% to 52%. In five studies, the prevalence of SLD was assessed in acromegaly patients irrespective of disease activity, i.e. total acromegaly (TA) and was reported to range from 31% to 78%, respectively [14–16, 22, 23].

The studies that assessed the prevalence of liver steatosis and intrahepatic lipid content are summarized in Table 1, including information on the study model, imaging techniques used, study population, prevalence of steatosis, and intergroup comparisons.

**Table 1:** Liver steatosis prevalence and intrahepatic lipid content in patients with acromegaly

Study	Study model	Examination technique	Steatosis liver disease reported	TA no. of patients included in the study	AA no. of patients assessed for liver outcome	CA no. of patients assessed for liver outcome	Prevalence of liver steatosis	Outcome group comparisons	Diabetes prevalence	Mean BMI [kg/m^2^]
Karadeniz [15]	Retrospective case-control	Liver USG	MASLD	58	24	26	AA 71% CA 92% TA 78% CG 18%	AA = CA TA > CG	AA: 33% CA: 23% TA: 29% CG: %	TA: 31 AA: 30 CA: 31 CG: 30
Arslan [21]	Retrospective cross-sectional	Liver USG	NAFLD	60	60*	–	AA 29% CG 42%	AA = CG	AA: NR CG: 0%	AA: 30 CG: 27
Bankir [17]	Cross-sectional	Liver USG	Liver steatosis	40	20	20	AA 45% CA 30% CG 20%	NR	AA: 40% CA: 20% CG: 0%	AA: 29 CA: 29 CG: 28
Coskun [18]	Prospective cross-sectional	Liver USG with TAI technique	NAFLD	23	23*	–	AA 26% CG 52%	AA < CG	AA: 35% controlledNIDDM CG: 0%	AA: 27 CG: 28
Yamamoto [19]	Retrospective cross-sectional	Liver USG	Liver steatosis and MASLD	58	58 *	–	AA 17.2% liver steatosis 15.5% MASLD	AA with BMI below 25 < AA with BMI greater than or equal to 25	IGT or DM: AA BMI < 25: 69% AA BMI ≥ 25: 85% TA: 77.2%	AA BMI < 25: 23 AA BMI ≥ 25: 28
Saleem [20]	Cross-sectional	Liver USG with CAP and liver biopsy	Liver steatosis	50	50* by USG 20* by biopsy	–	AA 70% (imaging) AA 65% (biopsy)	NR	AA: NR CG: 0%	NR
Eroglu [23]	Prospective cross-sectional	MRI-PDFF	NAFLD	32	15*	17	AA 0% CA 58.7% TA 31.3% CG 26.3%	AA < CA AA ≤ CG (*p* = 0.057) CA = CG (*p* = 0,09)	AA: 27% CA: 29% CG: 0% TA: 28% only NIDDM	AA: 30 CA: 32 CG: 29 TA: NR
Koutsou-Tassopoulou [14]	Cross-sectional	Liver USG with CAP	NAFLD	22 included 18 liver USG assessed	NR	NR	TA 67%	–	TA: 36%	TA: 31
Ciresi [16]	Prospective longitudinal	Liver USG	Liver steatosis	31	31*	18	AA 61.2% TA 61.2%	TA = AA	DM excluded	AA pre-treatment: 29 TA post-treatment:29
Apaydin [22]	Cross-sectional	Liver USG with CAP	MASLD	58	13	45	TA 50% CG 47% MMCG: NR	Moderate-severe liver steatosis TA< MMCG TA = CG	TA: 44% CG: 0% MMCG: 33% GLP1-RA or PGTZ treated excluded	AA: 31 CA: 30 TA: 30 CG:27 MMCG: 32
Fellinger [33]	Prospective cross-sectional	1 H-MRS	IHL	15	12 ^	–	–	AA < CG	AA: NR CG: 0%	AA 27 CG 27
Winhofer [30]	Prospective longitudinal/ cross-sectional	1 H-MRS	IHL	10	10 *	7	-	AA < CG CA = AA	DM excluded	AA 28 CG: 27
Szendroedi [28]	Prospective cross-sectional	1 H-MRS	IHL	7	–	7	–	CA > CG	TA: 0% CG: 0%	CA: 28 CG: 26
Bredella [24]	Prospective longitudinal/ cross-sectional	1 H-MRS	IHL	20	20 *	16	–	AA < CG CA = CG AA < CA	TA: 10% CG: 0%	AA: 30 CA: 31 CG: 31
Arlien-Søborg [32]	Prospective longitudinal/ cross-sectional	1 H-MRS	IHL	21	21 *	21	–	AA < CG AA < CA	AA = 11% CG = 0%	AA: 28 CA: 28 CG: 27
Kuker [27]	Prospective longitudinal/ cross-sectional	1 H-MRS	IHL	21	8	8	–	AA < CG AA < CA CA = CG	AA: 29% CA: NR CG: 0%	AA: 30 CA: 31 CG: matched to AA
Baumgartner [34]	Cross-sectional	1 H-MRS	IHL	6	4	–	–	–	–	–

AA- active acromegaly, BMI- Body Mass Index, CA- controlled acromegaly, CAP- controlled attenuation parameter, CG- control group, DM- diabetes mellitus, GLP1-RA- glucagon like peptide-1 receptor agonist, IGT- impaired glucose tolerance, IHL- intrahepatic lipid content, MAFLD- metabolic associated fatty liver disease, MASLD- metabolic dysfunction associated steatotic liver disease, MMCG- metabolically-matched controlled group, MRI-PDFF- magnetic resonance proton density fat fraction, NAFLD- non-alcoholic fatty liver disease, NIDDM- non-insulin dependent diabetes mellitus, NR- not reported, PGTZ- pioglitazone, TA- total acromegaly (controlled and active), TAI- Tissue Attenuation Imaging, USG- ultrasonography, 1 H-MRS- proton magnetic resonance spectroscopy, *- previously untreated AA patients, ^- active acromegaly without information about previous treatment, “-”- data irrelevant to the study model

Three studies compared the prevalence of SLD between AA and control groups [18, 21, 23]. Coskun et al., who utilized liver USG with TAI, found a lower prevalence of non-alcoholic fatty liver disease (NAFLD) in patients with AA than in control groups (17). Similarly, in a study by Eroglu et al. using MRI-PDFF, the authors reported a borderline significant lower prevalence of NAFLD in AA in comparison to controls (21). In contrast, Arslan et al., using conventional USG, did not find a statistically significant difference between the groups [21].

Only one study compared the prevalence of SLD, in detail NAFLD, between patients with CA and a control group. The difference was not statistically significant. In this study, MRI-PDFF was used; however, the number of patients that were included in the analysis was only fifteen [23].

Two studies compared the prevalence of SLD between patients with AA and patients with CA and the results were inconsistent [15, 23]. Eroglu et al., using MRI-PDFF, reported a higher prevalence of NAFLD in patients with CA [23], whereas in a retrospective study by Karadeniz et al., who utilized conventional USG, the prevalence of metabolic dysfunction-associated steatotic liver disease (MASLD) did not differ between these groups [15].

One study that assessed the prevalence of SLD, in detail MASLD, in TA reported no difference between acromegaly patients and matched controls [22].

Four studies assessed the prevalence of liver steatosis only in patients with acromegaly without a control group [14, 16, 19, 20], two of which evaluated only patients with AA [19, 20].

In the only prospective study that has assessed the prevalence of liver steatosis in patients with previously untreated AA. The percentage of patients with liver steatosis remained unchanged despite twelve months of pharmacological treatment with a somatostatin analog. However, 42% of the studied population had uncontrolled disease activity at the end of the study [16].

### Intrahepatic lipid content in patients with acromegaly

Several studies have assessed IHL content using proton magnetic resonance spectroscopy (1 H-MRS) without providing the proportion of patients with hepatic steatosis [24–34]. In the studies by Fellinger et al., Winhofer et al., Bredella et al., Arlien-Søborg et al., and Kuker et al., cross-sectional comparisons were performed between patients with previously untreated AA and a control group [24, 27, 30, 32, 33]. In all these studies, the IHL content was significantly lower in the AA groups than in the control groups.

In the studies by Szendroedi et al. and Bredella et al. [24, 28], IHL content was assessed in patients with CA and compared to that in the control groups. In the study by Szendroedi et al., the minimum duration of biochemical control among patients with CA was 7 years. In that cohort, the IHL content was three-fold higher in patients with CA than in the control group [28]. In contrast, Bredella et al. did not find a statistically significant difference in IHL content between CA and controls; however, the mean duration of acromegaly control in this study was only 8 months [24].

A subset of prospective studies evaluating IHL content have investigated the impact of treatment [24–27, 29–32]. Notably, some studies included both prospective and cross-sectional comparisons between study groups [24, 27, 30, 32]. The detailed findings from the prospective analyses are discussed in the section ‘*The influence of treatment modalities on liver steatosis and function’*.

### Prevalence of liver fibrosis in patients with acromegaly and intergroup comparisons

The risk of liver fibrosis was assessed using imaging techniques in four studies [14, 18, 20, 22], all of which had a cross-sectional design. All these studies used quantitative USG methods: two studies used shear wave elastography (SWE) [18, 20] and two studies used vibration-controlled transient elastography (VCTE) [14, 22]. In one study USG results were confirmed using liver biopsy [20].

To date, the two studies which assessed the prevalence of liver fibrosis in AA reported its rate at 13% and 40% [18, 20]. In two other studies that investigated liver fibrosis in TA cohorts the prevalence was reported at 29% and 5% [14, 22]. No study reported the prevalence of liver fibrosis in CA. The studies that assessed the prevalence of liver fibrosis are summarized in Table 2, including information on the study model, imaging techniques used, study population, prevalence of steatosis, and intergroup comparisons.

**Table 2:** Prevalence of liver fibrosis in patients with acromegaly

Study	Technique	TA (no. of patients)	AA (no. of patients assessed for liver outcome)	CA (no. of patients assessed for liver outcome)	Prevalence of liver fibrosis and intergroup comparisons	Diabetes prevalence	Mean BMI [kg/m^2^]
Coskun [18]	SWE	23	23*	–	AA > CG AA: 13% liver fibrosis 13% of patients exhibited fibrosis independent of steatosis	AA: 35% CG: 0% controlled NIDDM	AA: 27 CG: 28
Saleem [20]	SWE biopsy	50	50*	–	AA > CG (SWE based) AA: 40% liver fibrosis (biopsy based)	AA: NR CG: 0%	NR
Koutsou-Tassopoulou [14]	VCTE	22 included 17 USG assessed	NR	NR	TA: 29% liver fibrosis 6% liver cirrhosis	TA: 36%	TA: 31
Apaydin [22]	VCTE	58	13	45	TA: 5% MMCG: 15% CG:7% TA=MMCG = CG	TA: 44.1% CG: 0% MMCG: 33% GLP1-RA or PGTZ treated excluded	AA: 31 CA: 30 TA: 30 CG:27 MMCG: 32

AA- active acromegaly, BMI- Body Mass Index, CA- controlled acromegaly, CG- control group, DM- diabetes mellitus, GLP1-RA- glucagon like peptide-1 receptor agonist, MMCG- metabolically-matched controlled group, NIDDM- non-insulin dependent diabetes mellitus NR- not reported, PGTZ- pioglitazone, SWE- shear wave elastography, TA- total acromegaly (controlled and active), USG- ultrasonography, VCTE- vibration-controlled transient elastography, *- previously untreated AA patients, “-”- data not included in the study model

Among the two studies that compared the prevalence of liver fibrosis in AA and control groups both reported higher rates in the AA group [18, 20]. In the study by Coskun et al. among patients with newly diagnosed AA, 82.6% of patients had elevated liver stiffness and 13% showed evidence of liver fibrosis [18]. In the control group, elevated liver stiffness was observed in 52.2% of participants, while none had liver fibrosis. Notably, 13% of patients with AA exhibited fibrosis independent of steatosis. Saleem et al. reported that patients with AA exhibited a significantly higher liver stiffness measurement (LSM) than controls [20]. In the AA group, liver fibrosis was confirmed through biopsy in 40% of patients, consistent with elevated SWE measurements, which demonstrated a sensitivity of 88% and a specificity of 90%.

### Association of liver steatosis and liver function in acromegaly patients

Twelve studies assessed liver enzymes in acromegaly of which seven reported comparisons with a control group [14–20, 22, 23, 29, 31, 33]. The results are summarized in Table 3.

**Table 3:** Association of liver steatosis, fibrosis and liver function in acromegaly patients

Study	Assessed liver enzymes	Statistically significant differences	Differences in liver steatosis, IHL, stiffness and fibrosis between groups	Diabetes prevalence	Mean BMI kg/m^2^
Karadeniz [15]	AST, ALT, ALP, GGT, albumin, PLT	ALT: AA < CA AST, ALP: TA > CG albumin, PLT: TA < CG	Steatosis AA = CA TA > CG	AA: 33% CA: 23% TA: 29% CG: %	TA: 31 AA: 30 CA: 31 CG: 30
Bankir [17]	AST, ALT, ALP, GGT, PLT	ALP: AA > CG PLT: AA < CG	Liver stiffness AA > CA> CG	AA: 40% CA: 20% CG: 0%	AA: 29 CA: 29 CG: 28
Coskun [18]	AST, ALT, GGT, ALP	ALP: AA > CG	NAFLD: AA < CG Fibrosis: AA > CG	AA: 35% controlled NIDDM CG: 0%	AA: 27 CG: 28
Madsen [29]	ALT, ALP, bilirubin and coagulation factors (II, VII, X)	Increase of ALP, bilirubin and coagulation factors after adding PEGV to SSA (patients remained controlled at the beginning of the study)	PEGV + SSA increase in IHL	SSA only:17% SSA+ PEGV: 17%	SSA only 23 SSA+ PEGV: 24
Fellinger [33]	AST, ALT, GGT	NS	IHL: AA < CG	AA: NR CG: 0%	AA 27 CG 27
Yamamoto [19]	AST, ALT, GGT	NS AA with BMI < 25 vs. AA with BMI ≥ 25	Liver steatosis and MASLD AA with BMI greater than or equal to 25 > AA with BMI lower that 25	IGT or DM: AA BMI < 25: 69% AA BMI ≥ 25: 85% TA: 77.2%	AA BMI < 25: 23 AA BMI ≥ 25: 28
Saleem [20]	AST, ALT, PLT	AST, ALT: AA > CG PLT: AA < CG	Liver fibrosis AA > CG	AA: NR CG: 0%	NR
Eroglu [23]	AST, ALT	ALT: AA < CG	NAFLD: CA > AA AA ≤ CG (*p* = 0.057) CA = CG Liver stiffness AA = CA = CG	AA: 27% CA: 29% CG: 0% TA: 28% only NIDDM	AA: 30 CA: 32 CG: 29 TA: NR
Ciresi [16]	AST, ALT, GGT, ALP, bilirubin	No significant difference between AA group before and after 12 months of SSA treatment	Steatosis The same % of patients with liver steatosis before and after treatment	DM excluded	AA pre-treatment: 29 TA post-treatment: 29
Xie [31]	ALT, AST	Increase of ALT, AST post TSS surgical intervention	IHL increase	DM excluded	26
Koutsou-Tassopoulou [14]	ALT, AST, ALP, GGT	–	–	TA: 36%	TA: 31
Apaydin [22]	AST, ALT, GGT, INR, PLT.	ALT: TA < MMCG AST: TA < MMCG	Moderate-severe liver steatosis TA = CG TA< MMCG liver fibrosis: TA=MMCG = CG	TA: 44% CG: 0% MMCG: 33% GLP1-RA or PGTZ treated excluded	AA: 31 CA: 30 TA: 30 CG:27 MMCG: 32

AA- active acromegaly, ALT- alanine transaminase, ALP- alkaline phosphatase, AST- aspartate transaminase, BMI- Body Mass Index, CA- controlled acromegaly, CG- control group, DM- diabetes mellitus, GGT- gamma-glutamyl transferase, GLP1-RA- glucagon like peptide-1 receptor agonist, IGT- impaired glucose tolerance, IHL- intrahepatic lipid, INR- International Normalized Ratio, MASLD- metabolic dysfunction associated steatotic liver disease, MMCG-metabolically-matched controlled group, NAFLD- non-alcoholic fatty liver disease, NIDDM- non-insulin dependent diabetes mellitus, NR- not reported, NS- no significant difference, PEGV- pegvisomant, PGTZ- pioglitazone, PLT- platelets, SSA- somatostatin analogue, TA- total acromegaly (controlled and active), TSS- transsphenoidal surgery “-”- data irrelevant to the study model

In summary, four of seven studies pointed towards worse liver function in patients with acromegaly (including both controlled and active disease) when compared to controls [15, 17, 18, 20]. In three studies, changes in liver enzymes were correlated with hepatic steatosis [18, 22, 29]. In detail, Coskun et al., Apaydin et al. and Madsen et al. reported a significant association between alanine transaminase and hepatic steatosis measurement values [18, 22, 29]. Additionally Coskun et al. reported an association of hepatic steatosis and gamma-glutamyl transferase, while Mandsen et al. between IHL and alkaline phosphatase [18, 29].

Three studies evaluated the changes in liver function during specific treatment modalities, and their results are presented in detail in the following Section [16, 29, 31].

### The influence of treatment modalities on liver steatosis, intrahepatic lipid content and liver function

Eleven studies assessed the effects of treatment modality on hepatic lipid content and liver function in patients with acromegaly [14, 16, 22, 24–27, 29–32]. Nine studies were prospective [16, 24–27, 29–32], and two had a cross-sectional design [14, 22]. Eight studies assessed IHL content using 1 H-MRS [24–27, 29–32]; two used VCTE [14, 22], and one utilized conventional USG [16]. Overall, hepatic function in relation to treatment, assessed by liver enzyme levels, was reported in four studies [14, 16, 29, 31]. The results are summarized in Table 4.

**Table 4:** Effects of treatment modalities on IHL, liver steatosis and function in patients with acromegaly

Study	Study model	Liver imaging technique	Study participants	Therapeutic intervention	Outcome	Follow-up duration	Liver function	IHL/liver steatosis changes	Diabetes prevalence	Mean BMI kg/m^2^
Kuker [26]	Prospective /cross sectional	1 H-MRS	28*	TSS	28 AA → 26 CA/2AA	2.3 ± 1.3 years	–	IHL increase	DM excluded	Pre-surgery: 30 Post-surgery: 30
Madsen [29]	Prospective intervention study	1 H-MRS	18 CA on SSA divided into two groups	1) Group1 unchanged SSA monotherapy 2) Group 2 PEGV and SSA at reduces dose	18 CA → 18 CA	24 weeks	Group 2: increase in liver enzymes	Group 1 decrease in IHL. Group 2 increase in IHL.	SSA only:17% SSA+ PEGV: 17%	SSA only: 23 SSA+ PEGV: 24
Winhofer [30]	Prospective/cross-sectional	1 H-MRS	7 AA	TSS	7 AA → 7 CA	6 months	–	NS	DM excluded	AA 28 CG: 27
Bredella [24]	Prospective/cross-sectional study	1 H-MRS	16 AA	TSS: 15 patients, 1 cabergoline. Post-op: 5 patients on medical treatment	16 AA → 16 CA	8 months (range: 3–22)	–	IHL increase	TA: 10% CG: 0%	AA: 30 CA: 31 CG: 31
Reyes-Vidal [25]	Prospective	1 H-MRS	23 AA	TSS	follow-up	1 year	–	IHL increase		
Arlien-Soborg [32]	Prospective/cross-sectional	1 H-MRS	21 AA	TSS, post-op 11 patients on medical treatment	21 AA → 21 CA	44 weeks (range: 16–163)	–	IHL increase	AA: 11% CG: 0%	AA: 28 CA: 28 CG: 27
Xie [31]	Prospective/cross-sectional	1 H-MRS	17 AA	TSS	17 AA → 17 CA	1 year	Increase of ALT, AST	IHL increase	DM excluded	26
Kuker [27]	Prospective/cross-sectional	1 H-MRS	21 AA	PEGV treatment	21 AA → 21 CA	5.7 years (range: 1-13.4)	–	Increase in IHL on PEGV	AA: 29% CA: NR CG: 0%	AA: 30 CA: 31 CG: matched to AA
Ciresi [16]	Prospective	Liver USG	31*	SSA	31 AA → 18 CA /13 AA	12 months	NS	NS	DM excluded	AA pre-treatment: 29 TA post-treatment:29
Koutsou-Tassopoulou [14]	Cross-sectional	Liver USG with CAP	22 included 18 USG assessed	7 patients untreated 15 on medical treatment	18 CA	NR	–	NS	TA: 36%	TA: 31
Apaydin [22]	Cross-sectional	Liver USG with CAP	45 CA 13AA	15 TSS only; 30 TSS + SSA 1 gamma-knife	15 TSS: CA; 30 TSS + SSA: CA 1 gamma-knife: NR	median CA: 122 months AA: 87 months	ALT: TA < MMCG AST: TA < MMCG	CAP score AA = CA	TA: 44.1% CG: 0% MMCG: 33% GLP1-RA and PGTZ treated excluded	AA: 31 CA: 30 TA: 30 CG:27 MMCG: 32

AA- active acromegaly, ALT- alanine transaminase, AST- aspartate transaminase, BMI- Body Mass Index, CA- controlled acromegaly, CG- control group, DM- diabetes mellitus, GLP − 1- RA- glucagon like peptide-1 receptor agonist, IHL- intrahepatic lipid content, CAP- controlled attenuation parameter, NR- not reported, NS- no significant difference, *- previously untreated AA patients, MMCG- metabolically-matched controlled group, PEGV- pegvisomant, PGTZ- pioglitazone, SSA- somatostatin analogue, TA- total acromegaly (controlled and active), TSS- transsphenoidal surgery, USG- ultrasonography, 1 H-MRS- proton magnetic resonance spectroscopy, “-”- data irrelevant to the study model

Four studies included only surgically treated patients [25, 26, 30, 31]. Of these studies, one with an observation time of six months did not detect a statistically significant change in IHL content [30]. In contrast, three other studies, in which the observation time ranged from 12 months to a mean of 2.3 years, found a statistically significant increase in IHL content [25, 26, 31]. Notably, in the study by Xie et al., a decrease in IHL content was observed one month after surgery, followed by a gradual increase beginning at three months postoperatively [31]. Kuker et al. demonstrated that this trend persisted even 5 years after the intervention [26]. Interestingly it occurred despite a significant reduction of insulin resistance and absence of significant changes in BMI or waist circumference [26].

The effect of surgery on liver enzyme levels has been reported in a single study. Xie et al. demonstrated an increase in liver enzymes 12 months after the intervention, accompanied by a concurrent increase in IHL content [31].

Overall, the number of patients included in studies analyzing the effect of surgery on liver steatosis and function was relatively low, with the largest cohort comprising twenty-eight patients [26]. One study was also limited by the inclusion of patients who were not cured by surgery [26], and one did not report complete treatment outcomes [25].

Three studies included patients who were treated both surgically and pharmacologically [14, 24, 32]. Bredella et al. observed a gradual increase in hepatic lipids in patients with CA compared to AA before treatment [24]. The authors did not find a statistically significant difference between the patients who did and did not require medical therapy after surgery; however, this observation was limited by the small sample size (6 vs. 10 patients). Similarly, Arlien- Søborg et al. observed an increase in IHL content after treatment. However, in this study, the increase in IHL content was greater in patients who required medical therapy [32]. In a cross-sectional study, Koutsou-Tassopoulou et al. compared CAP scores in patients receiving pegvisomant with those not on medication and with those receiving somatostatin analogue (SSA) or cabergoline and found no differences between these groups [14]. Similarly, in a recent cross-sectional study, Apaydin et al. compared CAP scores in CA after transsphenoidal surgery with patients in biochemical control following SSA therapy, and likewise found no significant differences between the groups [22].

Three studies prospectively evaluated the effects of pharmacological therapy on hepatic function and lipid content [16, 27, 29]. As mentioned above, Ciresi et al. did not find a significant change in liver steatosis and function during treatment with a somatostatin analog [16]. Madsen et al. observed patients previously treated with somatostatin analog monotherapy [29]. These patients were randomized to either continue their therapy or receive co-treatment with pegvisomant and a reduced dose of a somatostatin analog for 6 months. The co-treatment group showed an increase in IHL content, followed by a simultaneous increase in alkaline phosphatase, bilirubin, and coagulation factors. In the group that continued the somatostatin analog treatment, IHL decreased. Similarly, in a subset of eight patients treated with pegvisomant, Kuker et al. found a gradual increase in IHL, which reached the level of the control group. The authors did not report any data on liver enzyme levels [27].

Four of the aforementioned studies analyzed the effects of pegvisomant treatment on liver steatosis [14, 27, 29, 32]. Three of these studies pointed towards an increase in IHL during GH receptor blockade [27, 29, 32], which, in one of the analyses, was found to be correlated with the weekly dose of pegvisomant [29].

## Discussion

In this scoping review we identified twenty-one studies that presented data on liver steatosis and fibrosis in acromegaly published between 2008 and 2026. The cohorts included patients with active and controlled disease who had undergone various treatment modalities. Liver outcomes were evaluated with the use of diverse radiological methods, indicating substantial heterogeneity among the studies. Nevertheless, the summarized data reveal a pattern of lower intracellular hepatic lipid (IHL) content in active acromegaly (AA) patients but a higher likelihood of liver fibrosis compared with controls. In line, the current data suggest that control of acromegaly may be associated with a rise in IHL content.

Radiological diagnosis of liver steatosis and fibrosis remains challenging. Ultrasonography (USG) is not specific and depends strongly on operator experience. Visualization of steatosis using USG is possible when more than 20% of the liver parenchyma is involved. However, USG can be enhanced using quantitative methods that enable non-invasive liver assessment. For example, shear wave elastography (SWE) or liver stiffness measurement (LSM) using vibration-controlled transient elastography (VCTE) are used for assessing liver stiffness, and the controlled attenuation parameter (CAP) or tissue attenuation imaging (TAI) quantify steatosis [35]. More advanced non-invasive techniques enabling the diagnosis of steatosis include computed tomography and magnetic resonance imaging (MRI). The highest accuracy is achieved using MRI proton density fat fraction (MRI-PDFF), the non-invasive gold standard for steatosis imaging, or proton magnetic resonance spectroscopy (1 H-MRS), which is increasingly replacing liver biopsy [36]. Moreover, MRI allows for the assessment of fibrosis using elastography; however, special hardware is required. Current guidelines allow the clinical diagnosis and treatment of metabolic dysfunction-associated steatotic liver disease (MASLD), but only liver biopsy provides a definite diagnosis of steatohepatitis [35].

Of the three studies that compared the prevalence of non-alcoholic fatty liver disease (NAFLD) in AA with that in the control group [18, 21, 23], two pointed towards a lower prevalence of liver steatosis in AA [18, 23]. The discrepancies among these results may be attributable to the differences in the diagnostic methods used. Coskun et al. employed USG with CAP [18] and Eroglu et al. used MRI-PDFF [23], both of which offer higher sensitivity for detection of hepatic steatosis than conventional ultrasound applied in the study by Arslan et al. [21]. Moreover, in all studies that assessed IHL using 1 H-MRS [24, 26, 30, 32, 33], the IHL content in the AA group was significantly lower than that in the control group. Of note, in the study by Winhofer et al. IHL in the AA group accounted for approximately 1% of the signal, which was unexpectedly low given the patients’ age and body mass index (BMI) [30]. In addition, the prevalence of hepatic steatosis among treatment-naive AA in the study by Yamamoto et al. was lower than the previously reported prevalence of 25.5% for NAFLD in the general Japanese population [19, 37].

In summary, the data presented in this literature review reveal a pattern of lower IHL content in AA patients compared to controls. Given the coexistence of insulin resistance and peripheral lipolysis caused by excess growth hormone (GH), the results of the above mentioned studies could be perceived as a paradox. However, these findings reflect results from experimental models that examined the tissue-specific actions of GH on hepatocytes. Vázquez-Borrego et al. used a mouse model of adult-onset hepatocyte-specific GH receptor knockdown and demonstrated the role of GH in reducing hepatic de novo lipogenesis [38]. In this study, mice with GH receptor knockdown developed severe liver steatosis. Interestingly, this pathological state was only partially reversed by viral vector-mediated expression of Stat5b in hepatocytes. This suggests that GH, independently of STAT5b and its downstream target insulin growth factor-I (IGF-I), alters the hepatic transcriptome to reduce glycolysis, de novo lipogenesis, and liver steatosis [38]. These experimental observations are further supported by recent human studies. Fellinger et al., in a group of metabolically healthy individuals, reported that IHL content was inversely associated with IGF-I levels and the GH nadir in an oral glucose tolerance test. The multicollinearity test revealed that these two parameters were independent factors [39]. More recently, Baumgartner et al. showed that, in healthy individuals, GH stimulates the secretion of very low density lipoprotein (VLDL) triglycerides, thus protecting against liver steatosis [34].

Based on the data mentioned above, a research question arises regarding the consequences of controlling AA on IHL content. Glucose metabolism may be restored in 23–58% of patients with acromegaly after successful pituitary surgery, however remains unchanged or may even worsen with pharmacological control [40]. Gatto et al. demonstrated that the intramuscular adipose tissue did not decrease after achieving remission of acromegaly [41]. Therefore, it appears probable that persistence of cardio-metabolic factors, along with the loss of the protective effect of high GH level on hepatocytes, may result in the accumulation of IHL. Indeed, this phenomenon has been observed in the retrieved prospective studies [24–26, 31, 32] as well as in one cross-sectional study comparing the prevalence of liver steatosis in patients with AA and controlled acromegaly (CA) using MRI [23]. The only exception among prospective studies was that by Winhofer, who evaluated AA patients six months after surgery and found IHL to be equal between the AA and CA groups [30]. The outcomes of this study may be explained by its relatively short observation period. The insignificant difference between CA and AA reported by Karadeniz et al. may be explained by the use of conventional USG in their study [15]. Furthermore, only Eroglu et al. evaluated patients with previously untreated acromegaly, thereby eliminating the potential influence of therapy on the results [23].

Whether the accumulation of IHL in patients with CA exceeds that in non-acromegaly matched individuals is not clear, as comparisons of individuals with CA to control groups with respect to hepatic steatosis have been limited. Only the study by Eroglu et al. assessed the prevalence of NAFLD in these patient groups [23]. Although NAFLD affected 58.7% of CA patients and 26.3% of control group patients, the difference did not reach statistical significance. Three other studies compared the IHL content using 1 H-MRS in the CA and control groups [24, 27, 28]. In a study by Szendroedi et al., in which patients in the CA group had documented at least seven years of biochemical remission, the IHL content was significantly higher in CA than in control groups [28]. In contrast, in studies the by Bredella et al. and Kuker et al., IHL content was similar between CA and control groups [24, 27].

The observed differences in the retrieved studies may also result from the different criteria used to define disease control across studies. GH and IGF-I discordance after acromegaly treatment is a common phenomenon in the clinical setting [42, 43]. Given the specific impact of GH on hepatic lipogenesis this aspect should be taken into account. However, its occurrence was not reported in any of the studies and only six required basal or suppressed GH levels as a criterion for defining acromegaly control. Consequently, the CA groups in the included studies presumably comprised patients with normal IGF-I but unsuppressed GH levels, which could have influenced outcomes.

Two studies reported a higher risk of fibrosis in AA than in the control group [18, 20]. Notably, in the study by Coskun et al., 13% of patients exhibited fibrosis independent of steatosis [18]. This is in line with experimental studies showing that GH and IGF-I induce fibrosis by increasing collagen synthesis and deposition outside the cellular overgrowth [44]. Additionally, increased GH levels have been reported to cause increased fibrosis with ductular reactions and cholangiocyte proliferation [45].

The impact of pharmacological therapy on IHL content in patients with acromegaly remains unclear, as only six prospective studies on this topic have been identified, of which only three observed patients exclusively receiving medical therapy. Nevertheless, GH blockade by pegvisomant resulted in an increase in IHL content in the majority of studies. Pegvisomant is a highly effective medication for uncontrolled acromegaly [46]. It has a positive effect on glucose metabolism by enhancing peripheral and hepatic insulin sensitivity and reducing basal lipolysis [47]. Therefore, the reason for the observed increase in the IHL levels remains unclear. This may occur due to the loss of the specific role of GH in reducing hepatic lipogenesis or the restoration of IHL to normal values after achieving disease control.

The majority of the included studies suggest the presence of altered liver function parameters in patients with acromegaly compared with control populations, irrespective of disease activity. Nevertheless, only a small subset of studies has explored potential associations between liver enzyme levels and hepatic steatosis or fibrosis, with results that appear limited and heterogeneous. In addition, relatively few studies have addressed changes in liver enzyme activity during pharmacological treatment, indicating that the available evidence in this area remains limited.

Body composition in acromegaly patients is gender specific and dependent on sex hormones. The impact of GH on fat mass has been reported in female patients only [48]. However, among the studies included in our review only two have assessed sex-related differences in hepatic steatosis, whereas none has reported such differences in liver fibrosis. In the studies by Reyes-Vidal et al. and Kuker et al., the increase in IHL was numerically greater in men than in women [25, 26]. However, the small study groups did not enable statistical comparisons.

In summary, the discrepancies noted between the studies included in this review may result from several factors, including the study groups, radiological methods applied, time of observation, and criteria for defining acromegaly control. Moreover, some studies assessed only the presence of steatosis, whereas others evaluated NAFLD or MASLD, with the diagnostic criteria for these conditions differing among studies. The limitations of this scoping review include the utilization of keywords rather than MeSH terms and performing the search in two databases.

Several research gaps emerged that warrant further high-quality studies. The studies varied substantially in their inclusion of diabetic patients in the study groups and controls. Consequently, the impact of diabetes on the outcomes is difficult to assess. Second, most studies evaluated patients regardless of whether they were in surgical remission or under pharmacological control. Finally, the influence of pituitary insufficiency has not yet been investigated in the cited literature. It is well known that patients with GH deficiency may have low-normal IGF-I levels. Consequently, patients who have undergone treatment for acromegaly may be at risk of under-recognized GH deficiency, either as a result of over-treatment or due to lack of awareness. This could act as an important confounding factor and potentially affect the results. An individualized approach to GH replacement therapy is required for patients who develop GH deficiency after successful treatment of acromegaly [49, 50]. Similarly, adrenal and gonadal deficiencies that could potentially affect IHL content were not investigated in the retrieved studies. Furthermore, the long-term effects of remission and potential sex-differences should also be considered.

In conclusion, high heterogeneity was observed among the studies, including the study models, imaging methods used, patient populations included, and outcomes. IHL content and hepatic fibrosis in patients with acromegaly may be influenced by disease control status and treatment modalities. Further high-quality studies are required to assess the risk of liver steatosis and fibrosis in this patient population.
